# Low-Intensity Resistance Exercise in Cardiac Rehabilitation: A Narrative Review of Mechanistic Evidence and Clinical Implications

**DOI:** 10.3390/jcm13237338

**Published:** 2024-12-02

**Authors:** Jemima Jansen, Paul W. Marshall, Jocelyne R. Benatar, Rebecca Cross, Tia K. Lindbom, Michael Kingsley

**Affiliations:** 1 Department of Exercise Sciences, University of Auckland, Auckland 1010, New Zealandjbenatar@adhb.govt.nz (J.R.B.);; 2 Greenlane Cardiovascular Service, Auckland City Hospital, Auckland 1023, New Zealand; 3 Department of Health Sciences, Macquarie University, Sydney 2113, Australia; 4 Holsworth Research Initiative, La Trobe Rural Health School, La Trobe University, Melbourne 3000, Australia

**Keywords:** cardiovascular disease, exercise, low intensity, frailty, sarcopenia

## Abstract

Cardiac rehabilitation, a multi-component intervention designed to mitigate the impact of cardiovascular disease, often underutilises low-intensity resistance exercise despite its potential benefits. This narrative review critically examines the mechanistic and clinical evidence supporting the incorporation of low-intensity resistance exercise into cardiac rehabilitation programmes. Research indicates that low-intensity resistance exercise induces hypertrophic adaptations by maximising muscle fibre activation through the size principle, effectively recruiting larger motor units as it approaches maximal effort. This activation promotes adaptation in both type I and II muscle fibres, resulting in comparable increases in myofibrillar protein synthesis and phosphorylation of key signalling proteins when compared to high-intensity resistance exercise. Low-intensity resistance exercise provides equivalent improvements in muscular strength and hypertrophy compared to high-intensity protocols while addressing barriers to participation, such as concerns about safety and logistical challenges. By facilitating engagement through a more accessible exercise modality, low-intensity resistance exercise might improve adherence rates and patient outcomes in cardiac rehabilitation. Additionally, the ability of low-intensity resistance exercise to address sarcopenia and frailty syndrome, significant determinants of cardiovascular disease progression, can enhance the recovery and overall quality of life for patients. This review establishes evidence-based recommendations for the inclusion of low-intensity resistance exercise in cardiac rehabilitation, offering a promising pathway to enhance the effectiveness of these programmes.

## 1. Introduction

Cardiac rehabilitation is a well-established and effective multi-component intervention designed to reduce the burden of cardiovascular disease on population health [1,2,3]. Since the first meta-analysis over three decades ago of ten cardiac rehabilitation trials, demonstrating a 24% reduction in all-cause mortality following myocardial infarction [4], comprehensive cardiac rehabilitation programmes have continued to evolve. These programmes, which combine exercise, patient education, and psychosocial support, have consistently shown improved survival, reduced risk of recurrent myocardial infarction, and enhanced exercise capacity [5,6].

Despite these recognised benefits, rates of patient enrolment and sustained participation within broader public health cardiac rehabilitation initiatives remain suboptimal around the world, with only 30 to 70% of eligible patients commencing rehabilitation [1,7]. Factors such as poor referral rates, travel difficulties, and under-resourced programmes likely contribute to this low uptake. Moreover, the increasing efficacy of medical treatments has raised questions about whether exercise-based cardiac rehabilitation offers additional benefits over standard post-discharge care (i.e., pharmacotherapy, routine follow-up appointments, general lifestyle advice [8]) for reducing mortality and readmission rates [9]. Notably, the most recent Cochrane review showed that while exercise-based cardiac rehabilitation results in a 23% reduction in cardiovascular mortality at follow-ups extending beyond 12 months, there was minimal impact on all-cause mortality and hospitalisation rates [5]. Given these challenges, alternative exercise modalities need to be considered that might be more feasible and effective for a broader patient population.

Resistance exercise, defined as physical activity involving muscular actions against external resistance, promotes a range of muscular, neural, and systemic adaptation [10]. The trainable variables typically associated with resistance exercise include skeletal muscle strength, power, hypertrophy, and endurance [11]. While aerobic exercise has traditionally dominated cardiac rehabilitation, evidence suggests resistance exercise can be safely incorporated into these programmes [12,13]. Additionally, resistance exercise offers similar benefits in attenuating cardiovascular disease risk factors [11,14]. Despite this potential, there remains a lack of information regarding the appropriate intensity of resistance exercise needed to elicit clinically meaningful improvements in cardiac rehabilitation [15].

Low-intensity resistance exercise, which has shown considerable efficacy in enhancing hypertrophy and strength in other populations over the past decade, represents a promising approach for cardiac rehabilitation. Low-intensity resistance exercise involves lifting lighter loads, typically between 30% and 40% of an individual’s one-repetition maximum (or 3–4 out of 10 using a Rating of Perceived Exertion scale), and performing repetitions to the point of near-maximal effort, often termed “repetition failure” (i.e., 20 to 25 repetitions per set with the last few repetitions reaching near-maximal effort). This approach contrasts with traditional resistance training, and resistance exercise prescription often observed within cardiac rehabilitation practice, which uses a combination of higher loads (i.e., ≥60% maximal strength) and fewer repetitions (i.e., less than 12 repetitions per set). Evidence demonstrates that multiple sets of low-intensity exercise offer similar benefits in strength and hypertrophy to lifting heavier loads (i.e., ≥60% maximal strength) [16,17]. In the context of cardiac rehabilitation, low-intensity resistance exercise presents a time-efficient and practical type of exercise for patients, especially those with limited exercise tolerance or frailty, as it induces broad physiological adaptations without excessive cardiovascular strain. Its versatility allows it to be performed with various equipment, such as resistance bands, light dumbbells, or bodyweight, making it adaptable for both clinical and home settings. By incorporating low-intensity resistance exercise into cardiac rehabilitation, patients can address musculoskeletal decline associated with frailty and sarcopenia while potentially enhancing cardiovascular function through reductions in systemic inflammation and improvements in vascular tone. Additionally, the practicality of low-intensity resistance exercise as a method of rehabilitation reduces common barriers to exercise adherence, including concerns about overexertion, injury risk, and time constraints. Consequently, low-intensity resistance exercise emerges as a promising adjunct or alternative to standard aerobic-based rehabilitation protocols, offering both functional and cardiovascular benefits.

This aim of this narrative review was to examine the mechanistic evidence and clinical efficacy of low-intensity resistance exercise in the context of cardiac rehabilitation and the concomitant effect on cardiovascular disease risk reduction. The specific objectives of this review were (1) to summarise the foundational mechanistic evidence for the historical use of aerobic exercise in cardiac rehabilitation, (2) to critically assess the mechanistic and training evidence supporting low-intensity resistance exercise as an adjunct or alternative to traditional aerobic-based interventions, and (3) to provide practical, evidence-based recommendations for integrating low-intensity resistance exercise into cardiac rehabilitation programmes, addressing gaps in current exercise prescription frameworks and highlighting its potential to deliver comparable clinical outcomes to conventional approaches.

## 2. Aerobic Exercise in Cardiac Rehabilitation

This section outlines the historical basis and evidence for aerobic exercise in cardiac rehabilitation, examining its mechanisms for mitigating cardiovascular risk factors and discussing clinical outcomes that demonstrate its efficacy in reducing hospital admissions and improving quality of life. Additionally, the challenges of low participation and adherence to cardiac rehabilitation programmes are described, highlighting the need for alternative strategies to enhance patient engagement.

### 2.1. Aerobic Exercise for Cardiovascular Disease Risk Factors

Aerobic exercise has long been recognised for its broad physiological benefits, which are particularly critical for individuals with cardiovascular disease due to the condition’s complex interplay with other health issues, such as metabolic syndrome, hypertension, and chronic inflammation [18,19]. These co-morbidities often impair cardiovascular and metabolic functions, including endothelial function, lipid metabolism, and autonomic regulation, heightening the risk of disease progression and adverse cardiovascular events [20,21]. Physiological adaptations from regular aerobic exercise counteract these factors and also contribute to improved neurohormonal activity, lipid profiles, and enhanced vascular tone, all of which reduce cardiovascular disease risk [22,23]. The remainder of this section provides an overview of the benefits of aerobic exercise for addressing key risk factors for cardiovascular disease.

Blood concentrations of high-density lipoprotein cholesterol (HDL-C), low-density lipoprotein cholesterol (LDL-C), and triglycerides influence cardiovascular risk. More specifically, high HDL-C concentrations indicate better cardiovascular health, whereas elevated LDL-C and triglyceride concentrations increase the risk of cardiovascular disease [24,25]. In addition, elevated total cholesterol concentrations (>200 mg/dL or 5.17 mmol/L) are associated with a twofold increase in cardiovascular disease risk [25,26]. Regular aerobic exercise has been shown to improve lipid profiles by raising HDL-C and lowering LDL-C and triglycerides, with higher-intensity exercise yielding greater effects, indicating a dose–response relationship between energy expenditure and lipid profile improvements [20]. Long-term improvements in HDL-C, LDL-C, triglycerides, and cholesterol from aerobic exercise have been observed in various cardiac rehabilitation cohorts, underscoring the clinical relevance of these lipid profile changes in disease management and prevention [27,28].

Atherosclerosis progression is another major risk factor for cardiovascular disease, with inflammation playing a central role in the development of atherosclerotic plaques [29]. Aerobic exercise mitigates inflammation, with improved lipid profiles and reductions in inflammatory cytokines contributing to enhanced endothelial function [22]. This also includes a reduction in cell adhesion, molecule expression, and macrophage activity, key contributors to foam cell formation and plaque development [22]. In addition, aerobic training promotes the release of nitric oxide (NO) from endothelial cells, which reduces the expression of inflammatory factors [22] and lowers serum levels of high-sensitivity C-reactive proteins, a critical inflammatory marker in atherosclerosis progression [29]. Aerobic exercise also leads to improved vascular tone, as endothelial dysfunction, characterised by impaired NO release and reduced vasodilatory capacity, is common in chronic heart failure. Regular physical activity can restore endothelial function by increasing blood flow and NO secretion [30]. Improved endothelial function has been measured following aerobic exercise-based cardiac rehabilitation programmes for patients post-acute myocardial infarction, those with stable cardiovascular disease, and patients with chronic heart failure [31,32]. This restoration of endothelial function mitigates atherosclerosis progression, as enhanced NO release and improved vascular tone can significantly lower inflammation and reduce the risk of cardiovascular events.

The benefits of aerobic exercise further extend to the reduction in systemic vasoconstriction, hypertension, and atherosclerosis-related processes [14,21]. Aerobic exercise reduces the production of angiotensin, which decreases vasoconstriction, water and sodium retention, and aldosterone production [23,33]. These adaptations help decrease sympathetic tone and improve parasympathetic activity through the modulation of plasma adrenomedullin and natriuretic peptides, which further enhance endothelial function [22]. These physiological adaptations collectively contribute to a notable reduction in systemic vasoconstriction and blood pressure, effects that are consistently observed in patients following aerobic-based cardiac rehabilitation programmes [21,22,34].

Platelet activation plays a significant role in the pathogenesis of unstable angina and myocardial infarction, as platelets release growth factors and inflammatory markers [35,36]. Aerobic exercise improves platelet function, providing an antithrombotic effect [37]. Although acute maximal-intensity exercise can increase platelet activation and aggregation markers such as platelet factor 4 and p-selectin, regular physical activity reduces platelet activation in response to subsequent acute exercise [27,35,37]. Consequently, aerobic exercise lowers the risk of major vascular thrombotic events [38], underscoring its protective role in cardiovascular health. Aerobic exercise, particularly when performed with additional high-intensity bouts, has been shown to induce significant reductions in platelet aggregation propensity in patients with cardiovascular disease [39], potentially reducing thrombotic risk.

Neurohormonal activation is an adaptive response to cardiac injury aimed at maintaining organ and tissue perfusion [40]. Increased neurohormonal activation is primarily driven by heightened sympathetic nervous system activity and the activation of the renin–angiotensin–aldosterone system (RAAS) [23,33], which results in elevated expression levels of vasopressin and atrial natriuretic peptide. However, prolonged neurohormonal activation can contribute to the progression of heart failure symptoms [40,41], mediated by vascular and cardiac remodelling [22]. Exercise training has been shown to counteract these detrimental changes in vascular and cardiac structure through multiple mechanisms [18,21]. Exercise training enhances parasympathetic tone [42], promoting angiogenesis and improved heart rate variability, contributing to favourable cardiac remodelling [43,44]. Additionally, exercise training can downregulate the activity of the RAAS, promoting increases in natriuretic peptide levels and decreases in sympathetic tone [23,33]. Aerobic exercise has been shown to improve various measures of neurohormonal activation in cardiovascular disease patients, including improved parasympathetic tone as measured from heart rate variability [45], reduced baroreflex sensitivity and muscle sympathetic nerve activity [46], and reductions in measures of the RAAS such as plasma angiotensin-II, aldosterone, and norepinephrine [33]. These reductions in neurohormonal activation contribute to the well-established reductions in blood pressure observed after aerobic-based cardiac rehabilitation [34].

### 2.2. Clinical Outcomes of Aerobic Exercise for Cardiac Rehabilitation

The chronic physiological adaptations that occur in patients following aerobic exercise translate effectively into clinical settings. Numerous studies provide robust evidence supporting the efficacy of aerobic exercise in cardiac rehabilitation. A meta-analysis of 47 randomised controlled trials involving 10,794 patients indicated that cardiac rehabilitation significantly reduces the overall risk of mortality following an ischaemic event, with a relative risk of 0.87 and an absolute risk reduction of 3.2% [47]. In addition to lowering mortality rates, cardiac rehabilitation has been associated with a reduced risk of overall hospital admissions for patients with heart failure, with a relative risk of 0.75 and an absolute risk reduction of 7.1% [48], as well as improvements in psychological well-being and quality of life. Notable enhancements have been reported in scores related to depression and anxiety [3]. These improvements are attributed to the relationship between structured exercise programmes and positive physiological effects, including enhanced endothelial function, reduced insulin resistance, lower inflammatory markers, improved blood pressure, and favourable fibrinolytic states [49].

Evidence supporting the safety of cardiac rehabilitation is substantial [5]. Participation in exercise rehabilitation does not increase the risk of all-cause mortality. For instance, one observational study reported only one cardiac event among 25,000 patients who engaged in 50,000 h of exercise training [50]. Additionally, another study documented one case of ventricular fibrillation per 111,996 patient-hours and one myocardial infarction per 294,118 patient-hours [51]. These findings underscore the relatively minor risk of experiencing an adverse event during rehabilitation.

### 2.3. Participation in Aerobic Exercise for Cardiac Rehabilitation

The mechanistic basis for aerobic exercise in cardiac rehabilitation is well-established [49]. Aerobic exercise targets numerous cardiovascular disease risk factors and promotes favourable adaptations. While the positive clinical outcomes of aerobic exercise in cardiac rehabilitation programmes reinforce its role in improving cardiovascular mortality rates and reducing hospital readmissions, significant disparities in participation and adherence to these programmes remain.

Participation rates in cardiac rehabilitation programmes are low, ranging from 19% to 34% [1,52]. Poor participation is associated with various factors, including lack of resources, funding, accessibility issues for ethnic minorities or lower socioeconomic classes, gender, age, comorbidities, disease perception, and education [3,53]. Low adherence rates are linked to factors such as psychological well-being, access to transportation, and enjoyment of the sessions [3]. Systematic analyses reveal that participation and adherence to cardiac rehabilitation decline with age and are significantly lower among women and patients with comorbidities [53]. Consequently, while the physiological and clinical benefits of aerobic exercise in cardiac rehabilitation are favourable, these benefits alone do not sufficiently promote participation.

Interventions are needed to provide similar physiological and clinical benefits to aerobic exercise while being more practical and applicable. Cardiac rehabilitation strategies that have been extensively explored include low-intensity, long-to-short-duration exercise, high-calorie expenditure, and high-intensity interval training. Although resistance exercise as a modality in cardiac rehabilitation has been less explored, evidence suggests its potential for similar outcomes as aerobic-based cardiac rehabilitation [11].

## 3. Resistance Exercise in Cardiac Rehabilitation

The role of resistance exercise in promoting positive adaptations, such as increased muscular strength, function, mass, endurance, and power, is well-established in healthy individuals. Its value in chronic disease populations, including cardiac patients, is increasingly recognised [54]. In cardiac rehabilitation, resistance training has demonstrated benefits across several outcome variables, such as improvements in body composition, increased muscle mass and strength, enhanced exercise capacity, and vascular homeostasis, all of which are associated with reduced cardiovascular disease risk [11,54]. Several studies have investigated the effectiveness of resistance exercise when combined with aerobic exercise or as a standalone intervention. Compared with aerobic training alone, a combined approach has been found to be more effective for improving fat-free mass, percentage of body fat, trunk fat, upper and lower limb strength [55,56], peak work capacity, and $\dot{V}$O_2peak_ [15,55]. Additionally, a meta-analysis of coronary artery disease patients reported that resistance training alone could improve exercise capacity, muscular strength, and $\dot{V}$O_2peak_ [57]. This section summarises the evidence for the general safety of resistance exercise in cardiac rehabilitation and overviews the primary effects of resistance exercise training programmes on key outcomes associated with successful rehabilitation.

### 3.1. The Safety of Resistance Exercise in Cardiac Rehabilitation

Although improvements in both cardiorespiratory fitness and muscular strength are needed to enhance physical functioning in cardiovascular disease patients [49], aerobic training remains the predominant prescription in cardiac rehabilitation, with resistance training often introduced only as a supplementary component during later stages [13]. Historically, aerobic exercise was preferred due to concerns over the safety of resistance training for this vulnerable population. It was assumed that in cardiovascular disease patients, static exercise could induce harmful increases in heart rate and arterial blood pressure, linked to elevated left end-diastolic pressure [58]. Additionally, resistance training after a cardiac event was thought to result in higher haemodynamic load, potentially leading to negative left ventricular remodelling [12].

However, a growing body of evidence now supports the safety of resistance exercise in cardiac rehabilitation, demonstrating similar positive outcomes to aerobic exercise [15]. For instance, weight training as early as four weeks post-myocardial infarction has been shown to be both safe and effective, resulting in meaningful improvements in $\dot{V}$O_2peak_ and muscular strength when combined with aerobic training [13]. Likewise, combined resistance and aerobic training has not been associated with negative remodelling of the left ventricle in cardiac rehabilitation patients [12].

### 3.2. Exercise Capacity

Peak oxygen consumption ($\dot{V}$O_2peak_) is a critical measure of cardiorespiratory fitness and an important target in cardiac rehabilitation. In patients with cardiovascular disease, $\dot{V}$O_2peak_ is a significant predictor of long-term survival [6]. While aerobic exercise is typically regarded as the superior modality for improving $\dot{V}$O_2peak_ [59], resistance exercise also demonstrates effectiveness in enhancing $\dot{V}$O_2peak_ when compared to usual care [57]. In patients with cardiovascular disease, resistance training does not appear to significantly alter cardiac output during rest or exercise. Instead, improvements in $\dot{V}$O_2peak_ are largely attributed to peripheral adaptations, such as enhanced oxygen extraction and utilisation in the working muscle [11]. Furthermore, evidence suggests that combining resistance training with aerobic exercise can yield even greater improvements in $\dot{V}$O_2peak_ for patients with cardiovascular disease [55].

### 3.3. Vascular Homeostasis

The vascular endothelium plays a critical role in regulating vascular homeostasis. Alterations in endothelial function contribute to the pathophysiology of atherosclerosis and are implicated in the development of thrombosis [60]. Endothelial dysfunction is commonly observed in patients with coronary atherosclerosis, acute and chronic myocardial ischaemia [61], and heart failure [62]. In patients with heart failure and coronary artery disease, this dysfunction often manifests as impaired flow-mediated dilation, which is associated with a poor clinical prognosis [62,63]. Aerobic exercise training has long been established as an effective non-pharmacological intervention to improve endothelial function in coronary artery disease patients [61]. More recently, research has highlighted the potential benefits of low-to-moderate-intensity resistance training in enhancing flow-mediated dilation as an estimate of endothelial function [64]. For example, a 4-week resistance exercise programme (3 days per week, 60–80% maximal strength exercises) improved brachial artery flow-mediated dilation in end-stage heart failure patients [62]. Likewise, a 4-week resistance training intervention (4 days per week, 60% maximal strength exercises) in patients following an acute myocardial infarction resulted in significant improvements in brachial arterial flow-mediated dilation [61]. These findings support the use of resistance training to enhance vasoreactivity in patients with heart failure and those recovering from an acute myocardial infarction.

### 3.4. Body Composition

In patients with cardiovascular disease, high muscle mass combined with low fat mass is associated with the lowest risk of cardiovascular-related mortality, suggesting that body composition plays a significant role in mitigating cardiovascular disease risk. Mechanistically, this relationship is likely mediated through improvements in insulin sensitivity, reductions in systemic inflammation, and a lower prevalence of metabolic syndrome [65]. Specifically, men with high body fat and low muscle mass are at a significantly increased risk of cardiovascular disease, with evidence linking this combination to an elevated 10-year cardiovascular risk [66]. While the association between body fat and cardiovascular risk is generally consistent across sexes [67], the protective effect of muscle mass against cardiovascular risk appears to be more pronounced in men [68]. This sex-specific relationship may reflect differences in muscle mass distribution, hormonal factors (e.g., testosterone), and other mechanisms through which muscle mass influences cardiovascular health [11,66,67].

Despite strong evidence connecting increased fat mass with a higher risk of cardiovascular mortality, limited studies have specifically explored the effects of resistance exercise on body composition changes in cardiac rehabilitation patients [54,55,56]. Most investigations have used combined exercise protocols, which demonstrate benefits in increasing total muscle mass of approximately 1.0 to 1.5 kg over 12 weeks of training in these patients.

### 3.5. Muscular Strength

Reduced muscle strength is a well-established, modifiable predictor of cardiovascular disease morbidity and mortality [69,70]. Greater grip strength, an indirect indicator of overall muscular strength, has been shown to correlate with a lower risk of all-cause and cardiovascular mortality, independently of adiposity [71]. Given its efficacy in improving muscular strength, resistance exercise is favoured in cardiac rehabilitation [54].

A meta-analysis demonstrated that resistance training significantly increased skeletal muscle strength in both the upper and lower extremities of middle-aged and elderly coronary artery disease patients [57]. Furthermore, combined resistance and aerobic training was found to elicit significantly greater improvements in upper and lower body muscular strength compared to aerobic training alone in coronary artery disease patients [55]. These findings underscore the role of resistance exercise in enhancing muscular strength and mitigating cardiovascular disease risk.

### 3.6. Lipid Profiles

Dyslipidaemia, characterised by elevated LDL cholesterol (LDL-C) and triglycerides along with reduced HDL cholesterol (HDL-C), is a significant risk factor for cardiovascular disease [20]. Elevated total cholesterol levels (>200 mg/dL or 5.17 mmol/L) have been linked to a two-fold increase in cardiovascular disease risk, while LDL-C levels exceeding 130 mg/dL are also associated with increased cardiovascular risk [24,26]. Aerobic exercise has been well-documented to improve lipid profiles, leading to favourable changes in lipoprotein concentrations, such as reductions in LDL-C and triglycerides [20,22]. Similar effects are seen with resistance exercise. A meta-analysis of resistance exercise interventions lasting longer than four weeks, with an average intensity of approximately 70% of one-repetition maximum, demonstrated significant reductions in total cholesterol, the ratio of total cholesterol to HDL-C, non-HDL-C, LDL-C, and triglycerides [72]. These changes were associated with a decreased risk of coronary cardiovascular disease, including a 5% reduction due to lowered total cholesterol, a 21% reduction in men related to improvements in the total cholesterol-to-HDL-C ratio, and a 3% to 7% reduction in triglycerides depending on sex [72].

### 3.7. Quality of Life

The aims of cardiac rehabilitation are to promote healthy lifestyle changes that reduce the diseases’ physiological and psychological effects [3,49]. The net benefit for patients is an improvement in quality of life, where resistance exercise has been shown to be beneficial in cardiac rehabilitation [55]. For example, systematic analyses have shown significant improvements in the quality of life of chronic heart failure patients following resistance training alone or when combined with aerobic exercise [73]. These improvements are attributed to resistance-exercise-induced improvements in muscle strength in chronic heart failure patients [74], as well as improvements in patient self-efficacy in performing their normal activity [73].

## 4. Resistance Exercise Intensity

While evidence supports the benefits of resistance exercise in cardiac rehabilitation, inconsistencies in programme design between and within studies make it challenging to define an optimal prescription. Careful consideration of resistance exercise intensity and the mechanisms driving adaptation is crucial to avoid common biases and misinterpretations of the evidence. In particular, it is essential to challenge the oversimplified view commonly observed in cardiac rehabilitation position statements and guidelines that strength outcomes can only be achieved with high-intensity resistance exercise (i.e., >80% of maximal strength), while endurance and strength are solely linked to low-to-moderate intensity [54]. This ‘heavier is better’ mindset, pervasive in strength and conditioning, limits a more nuanced understanding of how intensity and volume interact to shape training outcomes, presenting a barrier to broader applications of evidence-based resistance training principles.

Research examining resistance training in cardiac rehabilitation reveals a confusing interplay between training intensity and outcomes. For example, some results show that low-intensity resistance training (40% of one-repetition maximum) combined with aerobic training can significantly enhance muscular strength and $\dot{V}$O_2peak_ in patients with coronary artery disease and post-myocardial infarction recovery [13,56,75]. Moreover, low-intensity resistance muscle training during early rehabilitation contributes to increased HDL-C and a tendency towards greater lean tissue mass compared to aerobic training alone [76]. However, other studies show that while resistance training improves strength and endurance, the impact of training intensity is less clear, as variable-intensity resistance exercise protocols were used, which complicate direct comparisons [12,77,78,79,80]. Overall, while low- to moderate-intensity resistance training appears effective, the heterogeneity of protocols and intensities limits definitive conclusions regarding the optimal training intensity needed to maximise outcomes in cardiac rehabilitation.

More recently, a three-arm randomised controlled trial was conducted to compare the effects of low- and high-intensity resistance training combined with aerobic training versus aerobic training alone during early cardiac rehabilitation in patients with coronary artery disease [81]. Both resistance exercise interventions were progressive over 12 weeks, with 36 supervised sessions. The results showed that aerobic training combined with high-load resistance training produced greater improvements in $\dot{V}$O_2peak_ compared to aerobic training alone, but not when compared to low-load resistance training. High-load resistance training also elicited significantly greater knee extensor isometric strength gains (+17 ± 9%) compared to low-load resistance training (+10 ± 5%) and aerobic training alone (+1 ± 8%) [81]. In a similar trial using patients with chronic heart failure, low-load and high-load resistance training in combination with aerobic training yielded comparable improvements in $\dot{V}$O_2peak_ and muscle strength [82], although it has been argued there was an insufficient difference in the protocols with which to discriminate them [83].

These recent studies have formed the basis of discussion advocating for high-intensity resistance exercise prescription in cardiac rehabilitation [83]. However, issues in study design and interpretation complicate efforts to understand the efficacy of low-intensity resistance training. For instance, trials that equate training volume (i.e., the product of sets, repetitions, and load) across protocols undermine the fidelity of low-intensity resistance training [81,84], which relies on repetitions performed to task failure (i.e., 20 to 25 leg extension repetitions at 40% maximal strength) to induce adaptive responses [16,85]. Furthermore, claims of greater strength gains with high-intensity training in patients with cardiovascular disease, measured via maximal isometric contractions, are not always supported by other strength measures, such as the leg press one-repetition maximum within the same study [81].

Selective reporting of outcomes when comparing high- and low-intensity resistance exercise not only reflects a traditional bias favouring high-load training but also overlooks growing evidence that low-intensity resistance exercise can elicit comparable gains in strength and hypertrophy. Such biases highlight the need for a broader perspective, especially in clinical populations, where properly prescribed low-intensity resistance training (i.e., to failure across multiple sets) may offer a safer, more accessible alternative. These considerations underscore the importance of understanding the mechanistic basis for the efficacy of low-intensity resistance exercise, which will be discussed in the following section.

## 5. The Mechanistic Basis for Low-Intensity Resistance Exercise

Low-intensity resistance exercise to failure induces hypertrophic adaptations by maximising muscle fibre activation via the size principle, where motor units are recruited from low to high thresholds as effort increases [86]. While high-intensity exercise fully recruits motor units early, low-intensity exercise to failure engages larger motor units and optimises voluntary muscle activation [16,87], promoting adaptations in both type I and II fibres [88]. This muscle fibre recruitment results in comparable anabolic signalling and myofibril protein synthesis to high-load exercises [89,90], primarily mediated by the Akt-mTOR pathway, which is critical for achieving a positive net protein balance and thus hypertrophy [91]. Studies have shown similar hypertrophic outcomes between low-load and high-load resistance exercise when performed to failure, underscoring the efficacy of low-intensity protocols in stimulating muscle growth [17,92].

Muscle strength improvements arise from both hypertrophy (i.e., increased muscle mass) and neural adaptations, which enhance responsiveness in spinal and supraspinal pathways [93,94,95,96]. Evidence indicates comparable strength gains from low-load resistance exercises performed to failure. For example, Morton et al. [16] reported similar strength increases between high-repetition (30–50% of 1RM) and low-repetition (75–90% of 1RM) groups when both trained to failure, supporting previous findings that showed no differences in isometric strength gains across varying loads in untrained men [90]. While low-load resistance exercise can yield strength gains, current evidence generally favours high-load training for optimal strength adaptations [88,97,98]. Discrepancies between studies may be due to variations in programme design, where equated volume favours high-intensity training [84], and the types of exercises used in both the intervention and testing. Single-joint exercises, such as leg extensions, often show no differences in strength gains between loads [90], while multi-joint exercises, like the bench press, tend to favour higher loads [16]. To date, there is no conclusive evidence examining the mechanisms of adaptation that explain strength differences (or lack thereof) between low- and high-intensity resistance exercise programmes.

## 6. Proposal for Low-Intensity Resistance Exercise in Cardiac Rehabilitation

While resistance exercise is mechanistically supported in cardiac rehabilitation, it is still not widely adopted by patients and is predominantly prescribed as supplementary to aerobic exercise [5,99]. Historically, resistance exercise was considered unsafe for cardiac patients due to concerns about adverse haemodynamic responses, such as increases in heart rate, arterial blood pressure, and left ventricular remodelling [12,58]. Although these safety concerns have since been largely disproven, and the efficacy of resistance exercise has been confirmed [12,15], the initial apprehension might have led to a persistent fear of heavy lifting among patients, many of whom emphasise the importance of safety and are reluctant to engage in such activities [100].

In addition to concerns about safety, logistical barriers such as travel distance, transport availability, time constraints, and limited resources further impede participation in cardiac rehabilitation [3,53,101]. In this context, low-intensity resistance exercise performed to failure presents a promising alternative. It provides equivalent improvements in muscular strength and hypertrophy compared to high-intensity exercise, without the need for heavy weights. This approach could help reduce barriers to participation by providing a safer and more accessible exercise modality. Consequently, prescribing low-intensity resistance exercise may enhance engagement and improve outcomes in cardiac rehabilitation.

The following section explores the specific benefits of low-intensity resistance exercise, focusing on its potential to mitigate sarcopenia and frailty syndrome, two significant comorbidities that contribute to the progression of cardiovascular disease. These conditions contribute to multi-system decline, with sarcopenia leading to muscle loss and frailty resulting in functional impairment and reduced resilience. By facilitating hypertrophic and strength adaptations, low-intensity resistance exercise can enhance muscle mass and functional capacity without imposing excessive cardiovascular strain on patients [102]. Although a broad range of exercise programmes have been applied to improve outcomes in frail patients, adherence issues similar to those in cardiac rehabilitation have been documented [102,103]. Low-intensity resistance exercise, however, remains underutilised in this population, highlighting an opportunity to better address sarcopenia and frailty through targeted intervention.

### 6.1. Sarcopenia

Sarcopenia, the loss of muscle mass and strength, is a critical determinant of the pathophysiology and progression of cardiovascular disease [104] and is independently associated with increased mortality risk [105]. In addition to mediating exercise intolerance and ventilatory inefficiency, sarcopenia is linked with worsening clinical outcomes, poorer quality of life, and prolonged hospital stays among patients with cardiovascular disease [106]. The development of sarcopenia may be driven by several factors commonly associated with cardiovascular conditions, including hormonal changes, oxidative stress, physical inactivity, chronic inflammation, apoptosis, low muscle blood flow, endothelial dysfunction, malnutrition, and overactivation of the ubiquitin–proteasome system (UPS) [104].

At a physiological level, sarcopenia is characterised by the loss of skeletal muscle mass, often manifesting as atrophy of both type I and type II muscle fibres, decreased muscular capillary density, and fat infiltration [106]. Molecularly, sarcopenia is underpinned by alterations in the balance between protein synthesis and protein degradation. In healthy individuals, anabolic signals such as insulin-like growth factor 1 (IGF-1) activate the PI3K-Akt-mTOR pathway, promoting protein synthesis, suppressing protein degradation, and supporting muscle hypertrophy [104]. However, in cardiovascular disease, increased proteolysis often coincides with suppressed protein synthesis [105], contributing to muscle wasting.

Heart failure serves as a notable example of how cardiovascular disease can exacerbate sarcopenia. Patients with chronic heart failure commonly experience declines in anabolic hormones such as IGF-1 and growth hormone (GH) [104,106]. For instance, IGF-1 mRNA expression is significantly reduced in the skeletal muscle of patients with heart failure (>50% reduction) [105]. Given that IGF-1 and GH play essential roles in preserving skeletal muscle mass, these hormonal deficits contribute to impaired muscle function and reduced muscle mass in affected individuals [104]. Moreover, the UPS, a major regulator of protein degradation, is frequently upregulated in heart failure, with increased expression of components such as MuRF1, FoxO1, and FoxO3 [105], increasing muscle protein breakdown.

Disruption of the Akt/mTORC1 pathway and increased UPS activity contribute significantly to sarcopenia in patients with cardiovascular disease, highlighting the need for targeted interventions. Low-intensity resistance training may offer an effective treatment modality in cardiac rehabilitation, aimed at countering sarcopenia and its associated risks. Resistance exercise has been shown to modulate protein synthesis pathways and reduce muscle degradation; for example, a 4-week resistance training intervention led to downregulation of MuRF1 mRNA and atrogin-1 expression, decreased reactive oxygen species (ROS), and upregulation of OGF-1/Akt/ERK signalling, thereby mitigating muscle atrophy [107].

Overall, integrating low-intensity resistance exercise into cardiac rehabilitation programmes has the potential to address sarcopenia-related muscle loss, thereby improving functional capacity and reducing the burden of cardiovascular disease.

### 6.2. Frailty

Frailty syndrome, a condition characterised by a decrease in the reserve capacity of various biological systems in response to endogenous and exogenous stressors [108], often coexists with cardiovascular disease and triples the likelihood of its occurrence [109]. Frailty is associated with poor clinical outcomes [103], including higher rates of hospitalisation and mortality in cardiac patients [110]. Although sarcopenia is a risk factor for frailty syndrome, these conditions are distinct [111]; muscle alterations symptomatic of sarcopenia are only observed in approximately two-thirds of frail individuals. Frailty is characterised not only by decreased muscular strength and functional capacity but also by underlying disruptions at the molecular and physiological levels, involving neuromuscular, endocrine, and inflammatory pathways [112]. Similar to sarcopenia, frailty has been linked with declines in anabolic signalling, particularly reductions in IGF-1 and GH, which impair muscle protein synthesis and contribute to muscle weakness and decreased mobility [113].

Frailty is also associated with increased systemic inflammation [114], often termed ‘inflammaging’, characterised by elevated circulating levels of pro-inflammatory cytokines such as TNF-α, IL-6, and C-reactive protein (CRP) [115]. This chronic inflammatory state exacerbates catabolic processes, leading to accelerated muscle degradation and reduced regenerative capacity [116,117]. Low-intensity resistance exercise has been shown to attenuate inflammation by downregulating pro-inflammatory markers and enhancing the anti-inflammatory response, thereby reducing muscle catabolism [118,119,120]. Such adaptations may improve the anabolic–catabolic balance, mitigate frailty-related muscle loss, and enhance the overall functional reserve.

Furthermore, frailty-related declines in neuromuscular function often manifest as decreased motor unit recruitment and impaired neuromuscular activation, which can contribute to an increased fall risk and loss of independence [121]. Low-intensity resistance training may counter these deficits by promoting neuromuscular adaptation that improves strength, without imposing high cardiovascular demands [102,122]. These mechanistic insights support the use of low-intensity resistance exercise to target the physiological alterations associated with frailty and improve outcomes in cardiac patients at risk of frailty syndrome [103].

## 7. Practical Application

The worldwide implementation of cardiac rehabilitation programmes, particularly those focused on aerobic exercise, is challenged by low participation and adherence rates. Despite well-established benefits, including reductions in cardiovascular mortality and hospital readmissions, participation rates for cardiac rehabilitation remain between 19% and 34% [1]. Several factors contribute to poor participation, such as lack of resources, inadequate funding, accessibility issues affecting ethnic minorities and lower socioeconomic classes, and barriers related to gender, age, comorbidities, and disease perception [53,123]. Low adherence is also influenced by transportation difficulties, the psychological state of patients, and insufficient enjoyment of exercise sessions [3,7]. Participation and adherence tend to be lower among women, older adults, and patients with multiple health conditions [53,124].

Given these challenges, it is clear that the physiological benefits of a traditional, centre-based aerobic exercise cardiac rehabilitation programme alone are insufficient to ensure widespread participation in cardiac rehabilitation [7,125]. More feasible and accessible exercise modalities that can provide similar benefits are urgently needed [126]. While several strategies have been explored, including low-intensity prolonged exercises, high-calorie expenditure protocols, and high-intensity interval training, resistance exercise, particularly low-intensity resistance training, shows promise as an alternative.

Low-intensity resistance exercise has demonstrated comparable improvements in strength and hypertrophy to high-intensity resistance training and can target cardiovascular disease risk factors effectively, particularly the important comorbidities of sarcopenia and frailty. Implementing this form of exercise within cardiac rehabilitation programmes could enhance accessibility by reducing barriers such as the need for specialised equipment and perceived safety concerns. Incorporating low-intensity resistance exercises in home-based programmes may also offer a practical solution for patients with transportation difficulties and could promote higher adherence rates by providing a safe, effective, and flexible option.

Additionally, many patients do not tolerate the sweating and fatigue associated with traditional aerobic exercises, such as treadmill and stationary bike protocols [127]. For these individuals, low-intensity resistance training offers an alternative that could be prescribed at home that avoids these discomforts, potentially improving participation [127]. This is particularly relevant for certain cultural groups, such as Māori and Pasifika men in New Zealand, who may find strength-based activities more familiar and comfortable compared to aerobic exercises, which can feel foreign or disconnected from their experiences [128].

In summary, home-based cardiac rehabilitation incorporating low-intensity resistance exercises offers a promising strategy to enhance patient participation and adherence rates. As part of this review, a prototype programme was developed in collaboration with nurse specialists and patients from the Auckland Cardiac Rehabilitation Programme (see Supplementary Materials). This co-design approach ensured the exercise prescription was clearly communicated and easily understood by both healthcare providers and patients.

The programme was designed to be performed at home or in a hospital gym setting, two to three days per week. Given the absence of one-repetition-maximum testing in these environments, training intensity was guided by a 10-point Rating of Perceived Exertion (RPE) scale, with an initial load chosen at approximately 3 to 4 out of 10. The goal was to achieve near-maximal effort or repetition failure within 20 to 25 repetitions per set. After informal feedback from nursing staff and ten patients, the advice was refined to encourage starting with a load perceived as “easy” and continuing until it felt “really, really hard” (corresponding to RPE 3 and 9, respectively). Progressive overload principles were embedded within this framework, with patients advised to progress to multiple sets (up to 3 or 4) with one-minute rest intervals between sets. Advice was also provided within the programme, following feedback and input from patients, with regards to various options for altering and progressing the absolute load used (i.e., elastic bands, instruction on how to use bodyweight, use of fluid-filled bottles). Patient feedback also led to the inclusion of a ‘split’ training option, alternating muscle groups across days for variety, along with multiple exercise options for each muscle group. Interestingly, patients preferred booklet-based pictorial guides over online video demonstrations, citing practicality and ease of use.

Although this initiative was conducted as a service delivery project rather than a formal trial, it provided valuable insights into the programme’s feasibility. This model is now widely disseminated and utilised across New Zealand, Australia, and Oceania. A detailed example of how this home-based, low-intensity resistance exercise programme can be structured and implemented in cardiac rehabilitation is included in the Supplementary Materials.

### Future Research Directions

Further research should explore the long-term clinical outcomes of low-intensity resistance exercise in cardiac rehabilitation, including its effects on cardiovascular health, functional capacity, and patient-reported outcomes. Additionally, investigating the impact of low-intensity resistance exercise on frailty, sarcopenia, and psychological well-being could provide a more comprehensive understanding of its benefits. Comparative studies between low-intensity resistance exercise and traditional cardiac rehabilitation programmes would also clarify its relative efficacy, informing future guidelines and expanding its adoption in diverse healthcare settings.

Moreover, future research should consider monitoring the unique cardiovascular adaptations associated with resistance exercise compared to conventional aerobic-based cardiac rehabilitation [11]. This may provide better insight and therefore support for the widespread adoption of resistance exercise in cardiac rehabilitation practice. For example, resistance exercise imposes different myocardial loading patterns, potentially influencing myocardial efficiency, ventricular stiffness, and vascular tone [129]. Advanced echocardiographic techniques such as strain and myocardial work analysis could be valuable in capturing these distinct adaptations. Recent studies highlight the clinical potential of these methods for evaluating myocardial workload and function during cardiac rehabilitation [130,131], particularly in patients post-coronary artery bypass grafting. Integrating techniques such as these into future resistance-based cardiac rehabilitation studies could provide deeper mechanistic insights into the time course of adaptation, enhance personalised exercise prescriptions, and ultimately optimise clinical outcomes.

## 8. Conclusions

Resistance exercise, particularly at a low intensity, represents a viable and effective alternative that can safely target cardiovascular disease risk factors while being more practical for patients who face barriers to traditional rehabilitation. The mechanistic evidence supports the efficacy of low-intensity resistance exercise, showing comparable improvements in muscular hypertrophy and strength to those achieved through high-intensity training. Additionally, such interventions may offer an appealing solution to overcome logistical and psychological barriers that contribute to poor adherence, particularly when implemented in a home-based setting.

We propose that low-intensity resistance exercise is an appropriate next step in promoting participation and adherence in cardiac rehabilitation programmes. By integrating this approach, we aim to enhance accessibility, align with evolving patient needs, and ultimately support improved clinical outcomes for a broader patient population. Our collaborative development of a prototype programme within the Auckland Health District Cardiac Rehabilitation Programme supports this proof-of-concept, providing a foundation for future research and refinement. The practical model, designed based on patient feedback and feasibility considerations, represents a promising approach for enhancing the adoption of cardiac rehabilitation programmes.

## Supplementary Materials

The following supporting information can be downloaded at: https://www.mdpi.com/article/10.3390/jcm13237338/s1, Cardiac Home Resistance Exercise Programme.
