# Supplementary material for: Low-Intensity Resistance Exercise in Cardiac Rehabilitation: A Narrative Review of Mechanistic Evidence and Clinical Implications
Source: J Clin Med. 2024 Dec 2;13(23):7338. doi: 10.3390/jcm13237338 (PMC11642833; doi:10.3390/jcm13237338)
Supplement: Supplementary file 1 [file jcm-13-07338-s001.zip › jcm-3342112-supplementary.pdf]

Cardiac Home Resistance Exercise Programme

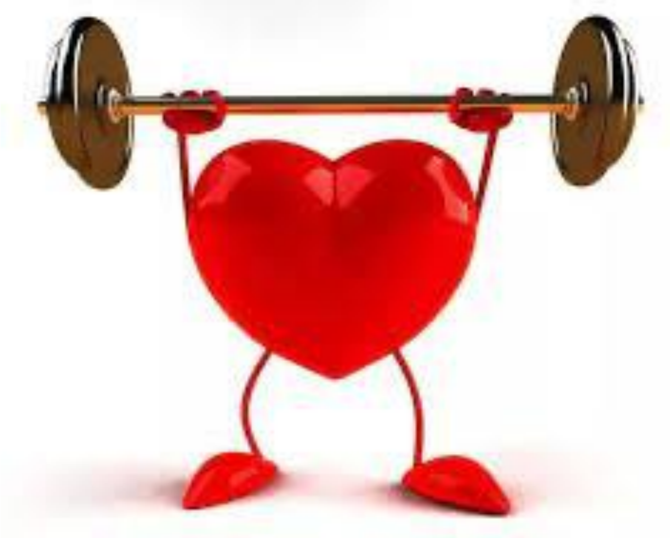

Name:

THE UNIVERSITY OF AUCKLAND
Te Whare Wānanga o Tāmaki Makaurau
NEW ZEALAND

SCIENCE
DEPARTMENT OF EXERCISE SCIENCES

Basic Programme Instruction

Why should you do resistance exercise? *It builds muscle which helps your heart and body health.*

What is my programme? *You have two different training days – one for the lower body, one for the upper body ☺ Do each programme once per week to start with. Your nurse will talk with you about exercise options, and show you how we will train your whole body.*

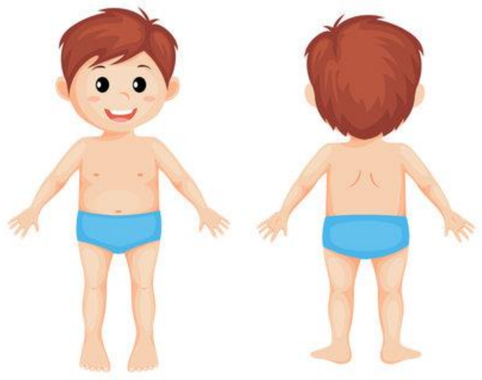

What do I need to do? *Pick an exercise for each part of your body (on each day). You want to add weight (if you need) so that the movement feels* ***EASY*** *the first time you do it. Then, keep doing the movement (called a repetition) until it feels* ***REALLY HARD****. Then you stop. Have a break for at least 1-minute, then repeat your set (if you can). Try and do ONE SET of each exercise the first time. If you go okay with that, try TWO SETS of each exercise the next time (and so on, up to THREE SETS of each exercise).*

How do I Add Weight (make it harder)? *Sometimes, your bodyweight will be enough. If the movement feels '****REALLY EASY'****, you can add weight. 1L of fluid = 1kg of weight, so if you have a 3L bottle (milk or juice – they have handles) you have up to a 3kg weight to use ☺ You could also buy a small set of dumb-bells, or a set of elastic bands (different colours = different 'weights' of band).*

THE GOAL IS TO PICK A WEIGHT THAT FEELS 'EASY' TO START WITH, AND TO LIFT IT UNTIL IT FEELS 'REALLY HARD'

Lower Body Day: _Pick one exercise for each muscle group_

Calf 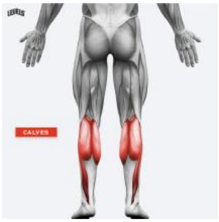	***Seated calf raise:*** *Raise heels and contract the muscle in the back of the leg.* 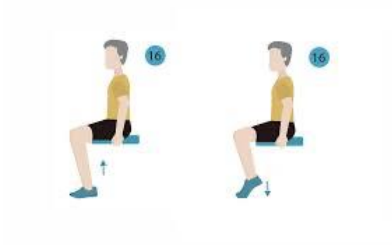	***Standing calf raise:*** *Raise heels, use a bench top for balance or a wall for support.* 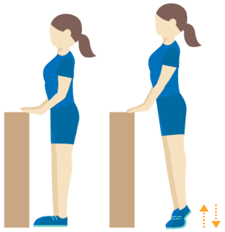	***Single Leg Calf Raise:*** *One foot at a time raise heel off ground, use a bench top for balance or a wall for support.* 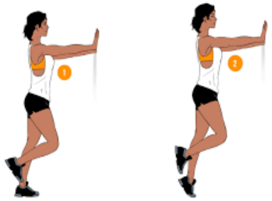
Quadricep 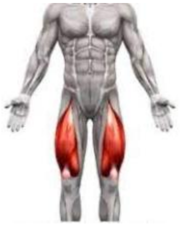	***Sit-to-stand:*** *Sit down slowly and with control on a sturdy chair, feet shoulder width apart, chest up, ensure knees move in line with toes, stand up.* 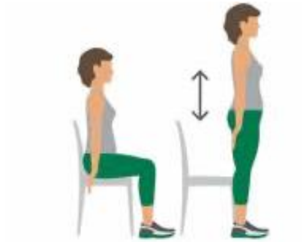	***Squat:*** *Squat down as if your sitting on a chair, chest up, brace core. Option to hold a weight at chest height to progress the movement.* 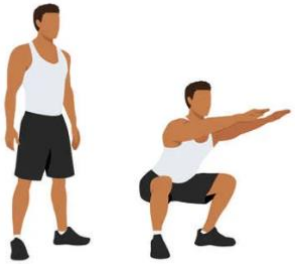	***Wall sit:*** *Aim to sit at knee height but start where is comfortable for you, core braced, lower back pressed to the wall.* 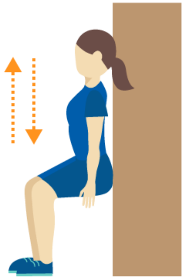
Gluteal and Hamstrings 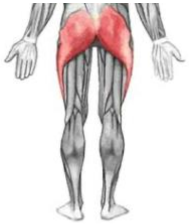	***Side and front raises:*** *Flex your foot to 90 degrees, extend leg backwards/side.* 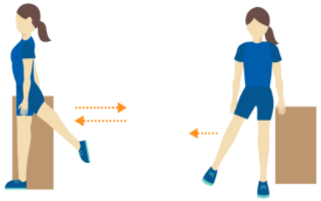	***Step ups:*** *Plant foot flat on step, drive your body up, control slowly back down.* 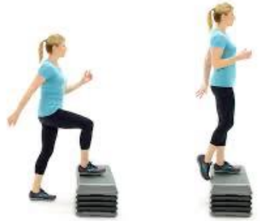	***Clams:*** *Lie on your side, feet together and in line with buttocks, start with knees on top of each other, while keeping feet together open and close top knee without twisting hips backwards.* 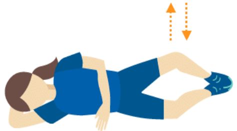

<u>Upper Body:</u> *<u>Pick one for each muscle group</u>*

<table>
<tr>
<td>Biceps
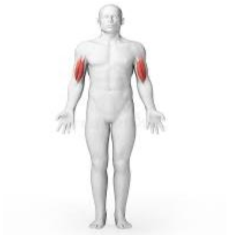</td>
<td colspan="2">Bicep Curls (seated/standing): Elbows tucked in by your side, exhale as you lift the weight up.
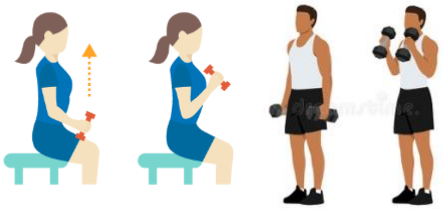</td>
</tr>
<tr>
<td>Triceps
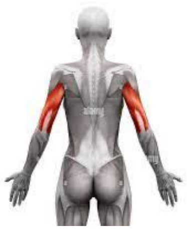</td>
<td>Triceps push backs: Tuck your elbow at your side, extend the elbow until it is straight and you feel the muscle in the back of your arm squeeze and contract. This exercise can be performed with one hand on top of the bench for support and slight bend forwards at your hips.
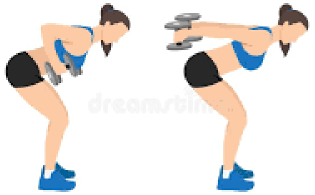</td>
<td>Triceps dips: Place hands on seat or bench, tuck elbows in, keep back straight, go as low as you can but not past your shoulders and then push up to fully extend at your elbows.
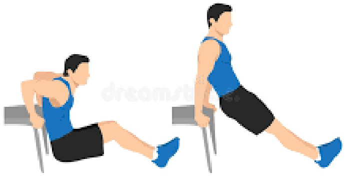
Note: The further your feet are away from you the harder it will be.</td>
</tr>
<tr>
<td>Shoulders
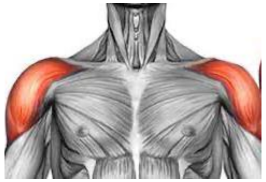</td>
<td>Seated overhead press: Elbows come down in line with your shoulders and then push the weight up above your head, fully extend at your elbow.
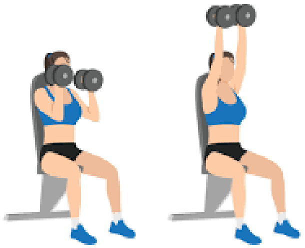</td>
<td>Standing lateral raises: Ensure core is strong, keep arms straight and raise arms to the side at shoulder height, slight bend through your elbow.
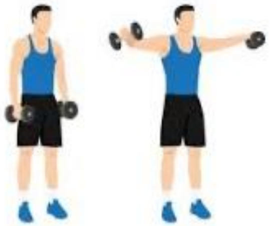</td>
</tr>
</table>

<table>
<tr><td>Chest
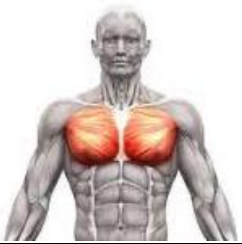</td><td colspan="2">Push Up: Hands just slightly wider than your shoulders, lower chest down to elbow height.
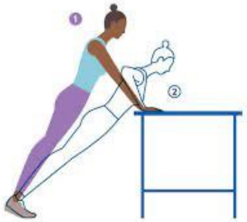</td><td>Chest Press: Lie on ground, elbows in line with shoulders, 90 degrees at elbow, press the arms up and fully extend at the elbow.
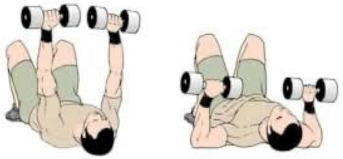</td></tr>
<tr><td>Back
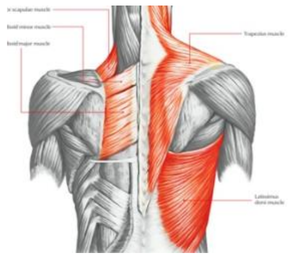</td><td colspan="3">Bent Over Row: Slight bend forwards at your hips, straight back, tuck your chin down to keep your back and neck straight, fully extend your arms and then row the weight towards your rib cage, squeeze your shoulder blades together at the top of the movement. This movement can be performed with one hand on a bench for extra support.
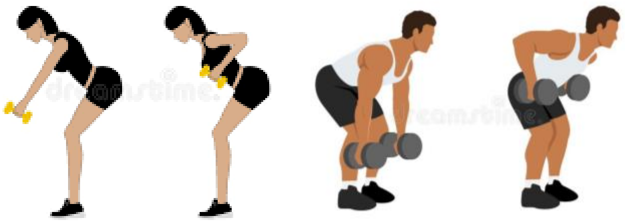</td></tr>
<tr><td>Trunk/Abs
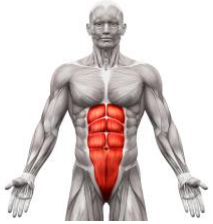</td><td>Marching on spot: Aim to lift your knees up to hip height.
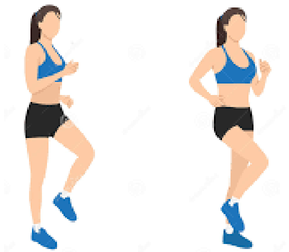</td><td>Dead bug: Tuck your hips under to press your lower back to the ground and engage your core, move opposite arm and leg to full extension without your lower back lifting off the ground.
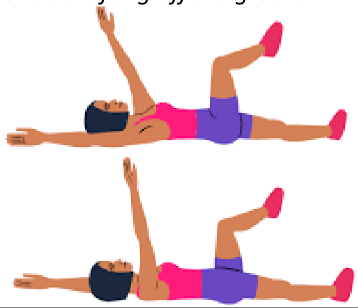</td><td>Plank Hold: You can be progress this movement starting by performing it with hands on a wall, shoulder width apart then on the ground on your knees or toes. Ensure you keep your back straight and remember to breath throughout the movement.
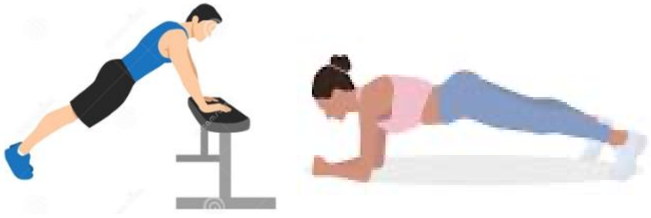</td></tr>
</table>

HOW HARD SHOULD I BE WORKING AGAIN? *Use this scale to judge the weight, remember, choose something that feels '**EASY'** and keep going until it feels '**REALLY HARD'**.*

Rating of Perceived Exertion (RPE Scale)	***What I should be doing***
Maximal	
Really, Really, Hard	***Finish the 'set' when the movement feels like this***
Really Hard	
Hard	
Challenging	
Moderate	***What the movement should feel like to start with***
Easy	
Really Easy	*Too Easy, Add a Bit of Weight*
Rest	

WHAT SHOULD I BE EATING WITH THIS TYPE OF PROGRAMME? *Talk with your nurse about what a healthy plate of food looks like. To 'gain muscle mass', all ages of people should be eating* ***PROTEIN****. Protein helps to build muscle, especially when combined with resistance exercise. Most people are* ***NOT EATING ENOUGH PROTEIN****.*

Here are some food options that will get you 10 grams of protein*. Over a day, you need to be eating (in grams) more than your bodyweight. So if you weight, 80kg, you need to be aiming for about 90 grams of protein per day.*

Animal Foods	Plant Foods
2 small eggs<br>30 g (1.5 slices) reduced fat cheese<br>70 g cottage cheese<br>1 cup (250 ml) low-fat milk<br>35 g lean beef, lamb or pork (cooked weight)<br>40 g lean chicken (cooked weight)<br>50 g grilled fish<br>50 g canned tuna or salmon<br>200 g reduced fat yoghurt<br>150 g light fromage frais	4 slices (120 g) wholemeal bread<br>3 cups (90 g) wholegrain cereal<br>2 cups (330 g) cooked pasta<br>3 cups (400 g) cooked rice<br>3/4 cup (150 g) lentils or kidney beans<br>200 g baked beans<br>120 g tofu<br>60 g nuts or seeds<br>300 ml soy milk<br>100 g soy meat
